# Simultaneous quantification of dasatinib, nilotinib, bosutinib, and ponatinib using high‐performance liquid chromatography–Photodiode array detection

**DOI:** 10.1002/jcla.24598

**Published:** 2022-07-12

**Authors:** Yuta Yokoyama, Eiji Nozawa, Miho Morita, Emi Ishikawa, Takehiko Mori, Masatoshi Sakurai, Taku Kikuchi, Eri Matsuki, Rie Yamazaki, Keisuke Kataoka, Aya Jibiki, Hitoshi Kawazoe, Sayo Suzuki, Tomonori Nakamura

**Affiliations:** ^1^ Division of Pharmaceutical Care Sciences, Center for Social Pharmacy and Pharmaceutical Care Sciences Keio University Faculty of Pharmacy Tokyo Japan; ^2^ Division of Pharmaceutical Care Sciences Keio University Graduate School of Pharmaceutical Sciences Tokyo Japan; ^3^ Division of Hematology, Department of Medicine Keio University School of Medicine Tokyo Japan; ^4^ Department of Hematology Tokyo Medical and Dental University Tokyo Japan; ^5^ Center for Transfusion Medicine and Cell Therapy, Department of Medicine Keio University School of Medicine Tokyo Japan

**Keywords:** chronic myeloid leukemia, high‐performance liquid chromatography, photodiode array detector, tyrosine kinase inhibitor

## Abstract

**Background:**

Dasatinib, nilotinib, and bosutinib, second‐generation tyrosine kinase inhibitors (TKIs), and ponatinib, a third‐generation TKI, are approved pharmaceuticals used in the treatment of chronic myeloid leukemia (CML). Although liquid chromatography‐tandem mass spectrometry assays for simultaneous quantification of the four TKIs in human serum have been reported in the literature, a high‐performance liquid chromatography (HPLC) assay that simultaneously quantifies these compounds has not yet been developed. This study aims to establish and validate an efficient HPLC analytical method using a photodiode array (PDA) detector for the simultaneous quantification of the four TKIs.

**Methods:**

Calibration standards were prepared by serial dilution of serum samples containing the four TKIs, followed by solid‐phase extraction. The four TKIs were eluted in order within 10 min using a binary HPLC gradient system.

**Results:**

The calibration ranges were 2–500 ng/ml for dasatinib, 100–5000 ng/ml for nilotinib, and 10–500 ng/ml for bosutinib and ponatinib. Intra‐day and inter‐day precision and accuracy values were found to be in accordance with the U.S. Food and Drug Administration guidelines. The recovery rates were 92.9%–96.0%, 80.7%–86.1%, 91.6%–99.0%, and 86.4%–92.6% for dasatinib, nilotinib, bosutinib, and ponatinib, respectively.

**Conclusion:**

To the best of our knowledge, this is the first report of an HPLC‐PDA analytical method that allows efficient simultaneous quantification of the four TKIs in the serum of patients with CML. We believe that the method developed herein can improve the efficiency of therapeutic drug monitoring in patients with CML in clinical practice.

## Graphical abstract

We established the first HPLC‐PDA analytical method for simple and rapid simultaneous quantification in serum concentration of dasatinib, nilotinib, bosutinib, and ponatinib. This HPLC‐PDA method can be applied to the TDM of the four TKIs for CML patients, enabling the construction of an optimal dosing regimen.

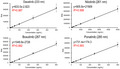

## 1 INTRODUCTION

Since 2001, when imatinib was introduced as a tyrosine kinase inhibitor (TKI), the prognosis of chronic myeloid leukemia (CML) has improved considerably; however, one‐third of patients still experience treatment failure because of developed resistance or intolerance to imatinib. To overcome this limitation, new‐generation TKIs with greater potency against BCR‐ABL1 kinase, and different safety profiles have been developed. Dasatinib and nilotinib, which are second‐generation TKIs, are effective against imatinib‐resistant or imatinib‐intolerant CML.^1^, ^2^ In 2010, these agents were used to treat newly diagnosed CML patients, achieving a higher optimal response rate and a lower risk of progression. Compared to imatinib, dasatinib, and nilotinib have shown 325‐fold and 25‐fold higher inhibitory effects against BCR‐ABL1, respectively; thus, they are expected to be as effective as imatinib as standard treatments for CML patients.^3^, ^4^ Use of bosutinib, another second‐generation TKI, is also an effective treatment option for patients who have developed resistance or intolerance to other TKIs. The major molecular response (MMR) rate was significantly higher in patients taking bosutinib, compared to imatinib.^5^, ^6^ Moreover, ponatinib, a third‐generation TKI, is effective in patients with acquired T351I‐mutated BCR‐ABL and those with resistance or intolerance to other TKIs.^7^

However, these TKIs are associated with frequent adverse events (AEs) such as pleural effusion when using dasatinib,^8^, ^9^ total bilirubin or lipase increase when using nilotinib,^10^ and diarrhea when using bosutinib.^11^ Such AEs are the most significant factors responsible for treatment discontinuation. In addition, a higher dose of ponatinib is associated with a higher incidence of AEs.^12^

The efficacy of therapeutic drug monitoring (TDM) for dasatinib and nilotinib treatment may depend on the association between drug concentrations in the blood and the therapeutic effect or AE development.^9^, ^13^ The treatment efficacy and AEs associated with other TKIs may also be correlated with the serum and plasma drug concentrations. If there is a correlation between drug concentrations in blood and drug efficacy, as indicated by MMR, cytogenetic response (CyR), and hematological response (HR), optimizing the dosing regimen could improve patient survival rate and quality of life. Therefore, the relationship between pharmacokinetic/pharmacodynamic parameters and the efficacy and safety of TDM in clinical patients with CML should be determined.

Although multicenter studies on liquid chromatography–tandem mass spectrometry (LC–MS/MS) assays for multiple TKIs have been conducted,^14^, ^15^, ^16^, ^17^, ^18^ clinical use remains challenging. In addition, high‐performance liquid chromatography (HPLC) assays that quantify each TKI separately have already been reported in literature.^14^, ^19^, ^20^, ^21^, ^22^, ^23^, ^24^, ^25^ Efficient simultaneous quantification of second‐generation and later‐generation TKIs using HPLC with photodiode array (PDA) detectors would improve TDM; however, such a method for dasatinib, nilotinib, bosutinib, and ponatinib has not yet been developed.

This study aims to establish and validate an HPLC‐PDA analytical method for the simultaneous quantification of the four TKIs. The method was used for serum concentration analyses of samples obtained from patients with CML.

## 2 MATERIALS AND METHODS

### 2.1 Chemicals and reagents

Dasatinib (98.44%), nilotinib (99.77%), and ponatinib (98.96%) were obtained from ChemScene; bosutinib (≥98%) was obtained from Cayman Chemical; HPLC‐grade acetonitrile was obtained from Merck KGaA; and potassium dihydrogen phosphate, phosphoric acid, HPLC‐grade methanol, and isopropanol were obtained from Nacalai Tesque. Pooled human serum was obtained from Cosmo Bio.

### 2.2 HPLC settings

An integrated HPLC system with PDA detector LC‐2040C 3D Plus (Shimadzu Corporation) was used with a Cadenza CX‐C18 analytical column (3.0 μm, 250 mm × 3 mm, IMTACT, Japan). The column temperature was 40°C. The injection volume of the prepared sample was 20 μl. The autosampler temperature was 4°C. Mobile phase A was 0.5% KH_2_PO_4_ (pH 3.5) – methanol (80:20). Mobile phase B was acetonitrile–methanol (80:20). The flow rate was 0.5 ml/min. The binary gradient program was 30%–70% B at 0–3 min, held 70% B at 3–4.5 min, 70%–30% B at 4.5–4.51 min, held 30% B at 4.5–10 min. The ultraviolet detection wavelengths for PDA were 233 nm for dasatinib, 261 nm for nilotinib, 267 nm for bosutinib, and 285 nm for ponatinib. The wavelengths were individually set by considering the maximum absorbance wavelengths and the suppression of the influence of interfering components.

### 2.3 Calibration standards and quality controls

Stock solutions for the four TKIs were mixed and diluted to a concentration of 0.5 mg/ml, prepared in methanol, and then diluted with methanol to a concentration of 50 μg/ml. The solutions were stored at −80°C. Calibration standards were prepared using blank human serum at the following concentrations: 2, 5, 10, 25, 50, 100, 250, and 500 ng/ml for dasatinib; 10, 25, 50, 100, 250, and 500 ng/ml for bosutinib and ponatinib; and 100, 250, 500, 1000, 2500, and 5000 ng/ml for nilotinib.

For quality control (QC), four concentration levels for the TKIs were prepared: (1) lower limit of quantification (LLOQ), (2) within three times the LLOQ (low QC), (3) medium‐range (medium QC), and (4) high‐range (high QC). The concentrations were 2, 5, 100, and 500 ng/ml for dasatinib; 10, 25, 250, and 500 ng/ml for bosutinib and ponatinib; and 100, 250, 2500, and 5000 ng/ml for nilotinib corresponding to (1), (2), (3), and (4), respectively.

### 2.4 Sample extraction

The four TKI analytes were extracted from human serum using a solid‐phase extraction (SPE) cartridge. Four TKI‐spiked serum samples (400 μl) were added to 600 μl of 2% phosphoric buffer solution and vortexed for 5 s. This mixture was used for the SPE procedure with a Phenomenex Strata‐X (30 mg/1 ml) cartridge preconditioned with 1.0 ml of methanol and 1.0 ml of water. The cartridge was then washed with 1.0 ml of water and 1.0 ml of 60% methanol and the analytes were eluted with 100% methanol (1.0 ml). The eluate was evaporated to dryness under a gentle stream of nitrogen using an MGS‐3100B spray‐on concentrator (Tokyo Rikakikai Co. Ltd.). The residue was dissolved in 25 μl of methanol and vortexed for 30 s. Next, 25 μl of the mobile phase was added, and the solution was vortexed for 30 s. The final extract (50 μl) was transferred into a vial, and 20 μl of each sample was injected into the HPLC apparatus.

### 2.5 Analytical method validation

The method was validated in accordance with the U.S. Food and Drug Administration (FDA) guidelines for bioanalytical method validation.^26^

#### 2.5.1 Calibration curve

Calibration curves were generated for each analyte by plotting the peak area vs. the theoretical concentration for eight (for dasatinib) or six (for bosutinib, ponatinib, and nilotinib) nonzero calibration standards, as described in Section 2.3. The peak areas were obtained from measurements on six different days, and the standards were prepared daily. A linear equation was obtained using least‐squares linear regression with weighting factors of 1/*x*, 1/*x* ^2^, 1/*y*, and 1/*y* ^2^, and without weighting, where *x* is the analyte concentration and *y* is the analyte peak area. The weighting factor corresponding to the lowest total percentage relative error was then selected.

#### 2.5.2 Precision and accuracy

To evaluate precision and accuracy, intra‐day and inter‐day precision and accuracy values were determined by analyzing the QC samples at four concentration levels (LLOQ, low QC, medium QC, and high QC) in quintuplicates for 1 day and in triplicates for three different days. Accuracy (%) is calculated as the percent of the mean observed concentration divided by the nominal concentration. Precision (% CV) is calculated as the percentage of standard deviation divided by mean observed concentration.

The dilution integrity evaluation of each analyte was performed using serum samples with concentrations five‐fold higher than high QC. The serum samples of each analyte were diluted five‐fold using blank serum.

The limits of detection (LOD) and quantification (LOQ) for each analyte were determined as the lowest concentrations with signal‐to‐noise ratios of three and 10, respectively.

#### 2.5.3 Selectivity

To determine the selectivity of the analytical method, six blank human serum samples from six different lots were selected and spiked with each analyte, whose LLOQ levels were then analyzed.

#### 2.5.4 Extraction recovery

The extraction recovery of the four TKIs from serum by SPE was determined by comparing the observed concentrations of three of the processed QCs (low, medium, and high QC) with those of the extracted blank serum spiked to the corresponding concentrations.

#### 2.5.5 Stability

The stability experiments were performed at two QC levels (low QC and high QC, *n* = 3) for each analyte in the human serum under the following conditions: (1) freeze–thaw stability for three freeze–thaw cycles where in each cycle, the samples were frozen at −80°C for at least 12 h, and then thawed at ambient temperature for 10 min before another freeze cycle.; (2) autosampler stability, which was performed by reinjecting the processed QC samples after 24 h at 4°C.

### 2.6 Patient samples

Steady‐state serum concentrations for dasatinib, nilotinib, and bosutinib in 10 patients (15 samples) with CML were obtained between August 2020 and January 2021 and assayed using the validated analytical method. This study was approved by the ethics committees of Keio University School of Medicine and the Faculty of Pharmacy (Approval No. 20140223, 200612–4). To participate in the study, all patients were informed about the study and informed consent was obtained.

## 3 RESULTS

### 3.1 Chromatography

**FIGURE 1:**
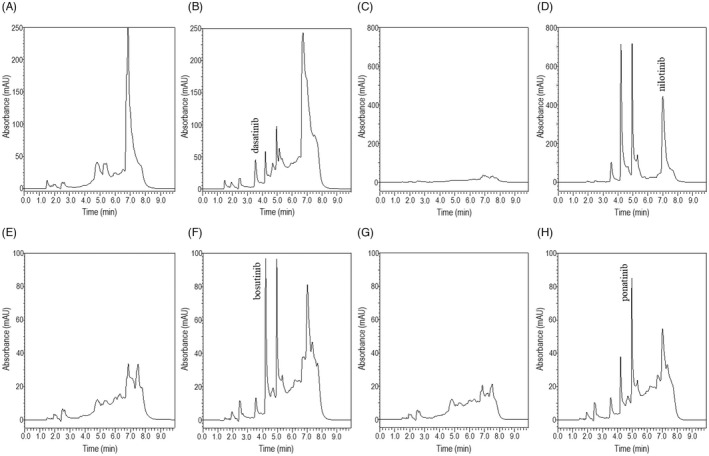
Representative chromatograms of extracts from (A) blank human serum (233 nm), (B) blank human serum spiked with 500 ng/ml dasatinib (233 nm), (C) blank human serum (261 nm), (D) blank human serum spiked with 5000 ng/ml nilotinib (261 nm), (E) blank human serum (267 nm), (F) blank human serum spiked with 500 ng/ml bosutinib (267 nm), (G) blank human serum (285 nm), and (H) blank human serum spiked with 500 ng/ml ponatinib (285 nm)

Representative chromatograms of blank human serum and blank human serum spiked with 500 ng/ml of dasatinib, bosutinib, and ponatinib, and 5000 ng/ml of nilotinib (Figure 1) were produced. There were no interfering peaks from the endogenous or exogenous compounds. Each of the four analytes showed no interference effect on the detection of the other TKIs. These four analytes were detected simultaneously within 10 min. The retention times for dasatinib, bosutinib, ponatinib, and nilotinib were 3.55, 4.21, 4.95, and 7.01 min, respectively. The additional peaks in each chromatogram that appear between the blank and the analyte derive from the other three analytes.

### 3.2 Calibration curves

**FIGURE 2:**
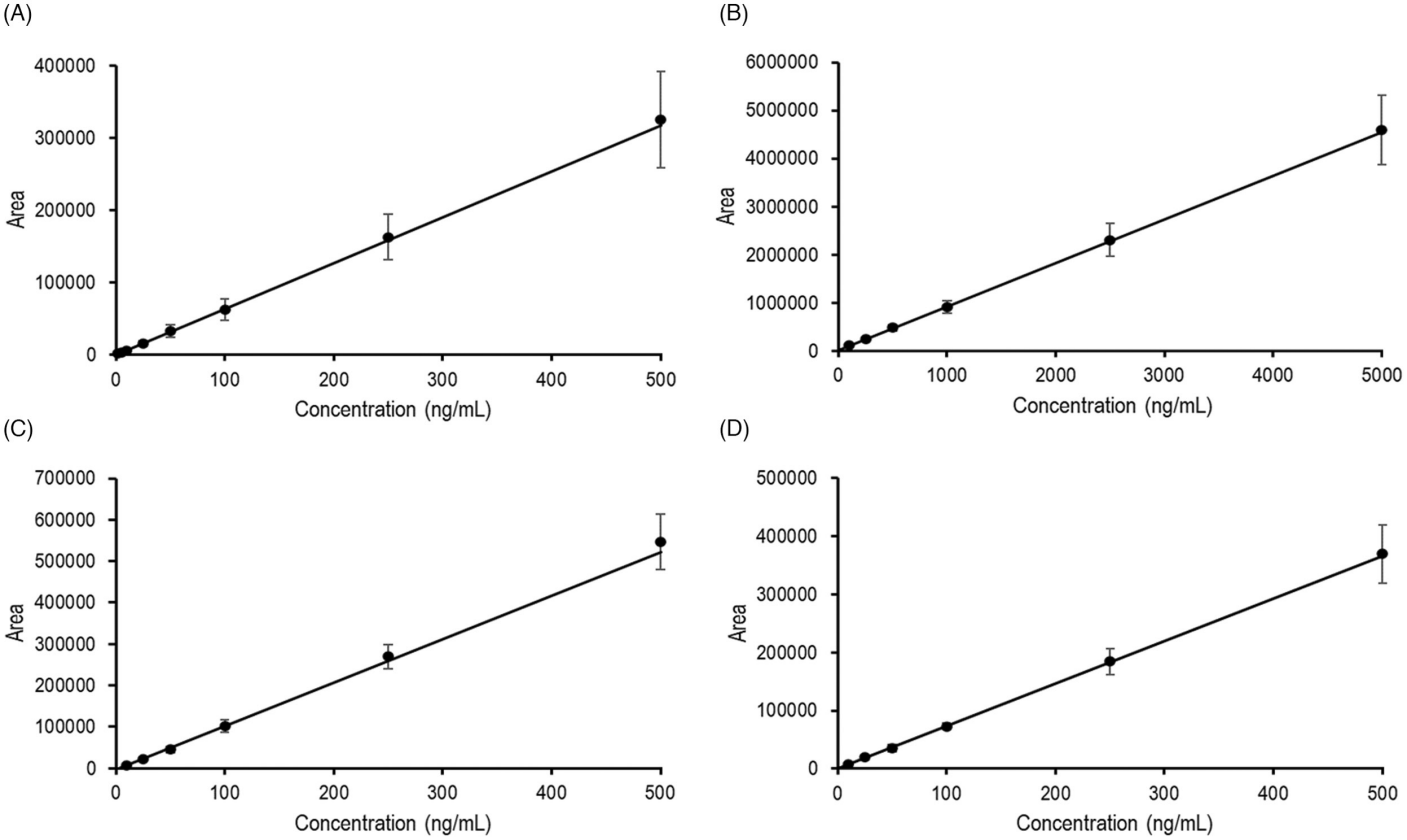
Calibration curves for (A) 2–500 ng/ml for dasatinib, (B) 100–5000 ng/ml for nilotinib, (C) 10–500 ng/ml for bosutinib, and (D) 10–500 ng/ml for ponatinib (*n* = 6)

The calibration curves were linear in the range of 2–500 ng/ml for dasatinib, 10–500 ng/ml for bosutinib and ponatinib, and 100–5000 ng/ml for nilotinib (*n* = 6) (Figure 2). A strong correlation was determined (*R* ^2^ ≥ 0.987 for the four analytes). The regression lines are presented in Table 1. The weighting factor was 1/*x* ^2^.

**TABLE 1:** Calibration data for dasatinib, nilotinib, bosutinib, and ponatinib (linear regression equation: *y* = ax + b) (*n* = 6)

	Regression line		Linearity (*R* ^2^)	Calibration range (ng/ml)	LLOQ (ng/ml)
	Slope, *a*	Intercept, *b*			
Dasatinib	633.0 ± 153.3	−2.8 ± 326.6	0.987 ± 0.006	2–500	2
Nilotinib	905.8 ± 128.0	21,909.2 ± 16,134.2	0.998 ± 0.001	100–5000	100
Bosutinib	1049.8 ± 141.5	−2728.3 ± 399.3	0.992 ± 0.008	10–500	10
Ponatinib	731.4 ± 95.9	174.3 ± 1983.0	0.993 ± 0.005	10–500	10

Abbreviation: LLOQ, lower limit of quantification.

### 3.3 Precision and accuracy

The intra‐day and inter‐day precision and accuracy values are summarized in Table 2. The intra‐day precision and accuracy values at LLOQ (*n* = 5) were within 20%. The precision values were within 9.0% and the accuracy values were within **±**14.4% for all the analytes. The intra‐day precision values and accuracy values at three QC levels (low QC, medium QC, and high QC) (*n* = 5) were within 15%. The precision values were within 10.1%, and the accuracy values were within ±12.6% for all the analytes. The inter‐day precision and accuracy values at LLOQ (*n* = 3) were within 20%. The precision values were within 17.9%, and the accuracy values were within ±13.8% for all the analytes. Those at three QC levels (*n* = 3) were within 15%. The precision values were within 13.7%, and the accuracy values were within ±6.3% for all the analytes.

**TABLE 2:** Intra‐day and inter‐day accuracy and precision for QC samples at four concentrations

Analyte	Level	Target concentration (ng/ml)	Intra‐day (*n* = 5)			Inter‐day (*n* = 3)		
			Average obtained concentratio*n* (ng/ml)	Precision (%)	Accuracy (%)	Average obtained concentration (ng/ml)	Precision (%)	Accuracy (%)
Dasatinib	LLOQ	2	2.3 ± 0.1	7.6	14.3	1.7 ± 0.3	17.9	−13.8
	Low QC	5	5.6 ± 0.2	6.0	12.6	5.1 ± 0.7	13.7	2.0
	Medium QC	100	100.8 ± 5.8	5.8	0.8	106.3 ± 2.7	2.5	6.3
	High QC	500	527.7 ± 20.9	4.0	5.5	502.7 ± 58.8	11.7	0.5
Nilotinib	LLOQ	100	85.6 ± 3.5	4.0	−14.4	90.2 ± 5.1	5.6	−9.8
	Low QC	250	226.7 ± 10.0	4.4	−9.3	234.9 ± 10.2	4.3	−6.0
	Medium QC	1000	967.6 ± 70.4	7.3	−3.2	958.2 ± 35.4	3.7	−4.2
	High QC	5000	5000.6 ± 196.9	3.9	0.0	5113.6 ± 63.0	1.2	2.3
Bosutinib	LLOQ	10	8.7 ± 0.3	3.9	−13.3	9.4 ± 1.2	12.8	−6.2
	Low QC	25	24.7 ± 1.6	6.3	−1.1	24.7 ± 1.3	5.4	−1.3
	Medium QC	250	248.6 ± 17.2	6.9	−0.6	249.4 ± 17.4	7.0	−0.3
	High QC	500	525.4 ± 12.8	2.4	5.0	521.5 ± 6.6	1.3	4.3
Ponatinib	LLOQ	10	9.7 ± 0.9	9.0	−2.7	8.8 ± 0.9	10.0	−11.9
	Low QC	25	26.8 ± 2.7	10.1	7.1	24.0 ± 1.6	6.7	−3.9
	Medium QC	250	259.8 ± 15.9	6.1	3.9	254.9 ± 10.9	4.3	2.0
	High QC	500	519.5 ± 29.6	5.7	3.9	499.8 ± 21.2	4.2	0.0

Abbreviations: LLOQ, lower limit of quantification; QC, quality control.

In the dilution integrity evaluation, CV values were below 6.4% and accuracy was 85.3%–99.0%.

The LODs for dasatinib, bosutinib, ponatinib, and nilotinib were 2, 16, 16, and 4 ng/ml, respectively, and LOQs were 2, 16, 40, and 16 ng/ml, respectively.

### 3.4 Selectivity

The average of the areas of six blank human serum samples from six different lots was <18.9% of the LLOQ of the analytes.

### 3.5 Extraction recovery

Recovery experiments for the four analytes were performed for the low, medium, and high QC concentrations. The mean extraction recoveries obtained using the SPE method were in the range of 92.9%–96.0%; the CVs were within 9.5% for dasatinib, 80.7%–86.1% and within 6.8% for nilotinib, 91.6%–99.0% and within 6.0% for bosutinib, and 86.4%–92.6% and within 10.0% for ponatinib, as shown in Table 3 (*n* = 4).

**TABLE 3:** Extraction recovery of each analyte of QC samples at three concentrations (*n* = 4)

Analyte	Level	Target concentration (ng/ml)	Extraction recovery (%)	
			Mean	CV
Dasatinib	Low QC	5	94.0 ± 8.4	9.0
	Medium QC	100	96.0 ± 6.7	7.0
	High QC	500	92.9 ± 8.8	9.5
Nilotinib	Low QC	250	86.1 ± 3.8	4.4
	Medium QC	1000	80.7 ± 4.8	5.9
	High QC	5000	83.3 ± 5.7	6.8
Bosutinib	Low QC	25	99.0 ± 5.1	5.1
	Medium QC	250	98.5 ± 3.0	3.1
	High QC	500	91.6 ± 5.5	6.0
Ponatinib	Low QC	25	87.9 ± 7.6	8.6
	Medium QC	250	86.4 ± 6.7	7.8
	High QC	500	92.6 ± 9.2	10.0

Abbreviations: CV, coefficient of variation; LLOQ, lower limit of quantification; QC, quality control.

### 3.6 Stability

Stability of the analytes was determined under two stability conditions for each analyte (Table 4). The degradation of each analyte in spiked serum after three freeze–thaw cycles and autosampler stability were within 14.5% and 4.6%, respectively.

**TABLE 4:** Stability of each analyte in the QC samples at two concentrations (*n* = 3)

Analyte	Level	Target concentration (ng/ml)	Freeze–thaw stability (%)	Autosampler stability (%)
Dasatinib	Low QC	5	104.5 ± 13.4	101.1 ± 6.9
	High QC	500	87.5 ± 2.7	103.8 ± 8.4
Nilotinib	Low QC	250	101.1 ± 6.6	107.5 ± 11.0
	High QC	5000	102.1 ± 2.0	109.3 ± 2.4
Bosutinib	Low QC	25	85.5 ± 7.8	95.4 ± 8.5
	High QC	500	92.5 ± 3.0	112.6 ± 14.8
Ponatinib	Low QC	25	103.7 ± 4.2	109.0 ± 6.6
	High QC	500	92.5 ± 3.4	110.3 ± 4.3

Abbreviation: QC, quality control.

### 3.7 Clinical application

The clinical application data for 10 patients (15 serum samples) with CML are summarized in Table 5. The serum concentrations for the TKIs at steady state (1.4–27.6 h after administration) in patients with a mean age of 46.9 ± 11.4 years (mean ± SD) were analyzed. Dasatinib (50–100 mg/day), nilotinib (600 mg/day), and bosutinib (400–600 mg/day) were analyzed in five, three, and two patients, respectively, and the measured concentrations were 5.4–140.8, 789.9–1897.3, and 92.4–299.9 ng/ml, respectively. In all the patients, the serum concentration of each analyte in the clinical sample was within the range of the calibration curve.

**TABLE 5:** Serum concentrations of TKIs in patients with chronic myeloid leukemia

Sample number	TKI	Dose regimen	Time after the last dose (h)	Serum concentration (ng/ml)
1	Dasatinib	100 mg QD	2.5	140.8
2	Bosutinib	400 mg QD	20.4	92.4
3	Nilotinib	600 mg BID^a^	15.7	1242.8
4	Dasatinib	100 mg QD	27.6	5.4
5	Bosutinib	600 mg QD	21.6	112.1
6	Dasatinib	100 mg QD	3.3	60.3
7	Dasatinib	100 mg QD	2.5	19.9
8	Nilotinib	600 mg BID^b^	4.4	954.9
9	Dasatinib	50 mg QD	3.0	103.2
10	Nilotinib	600 mg BID^b^	10.6	1503.9
11	Bosutinib	400 mg QD	2.4	299.9
12	Nilotinib	600 mg BID^b^	2.4	1897.3
13	Bosutinib	600 mg QD	9.4	275.0
14	Nilotinib	600 mg BID^b^	3.0	789.9
15	Nilotinib	600 mg BID^b^	1.4	1250.5

*Note*: QD, once daily; BID, twice daily.

^a^ 400 and 200 mg.

^b^ 300 and 300 mg.

## 4 DISCUSSION

To the best of our knowledge, this is the first report on an efficient HPLC‐PDA analytical method for simultaneous quantification of dasatinib, nilotinib, bosutinib, and ponatinib in human serum. Although several methods have been developed for the quantification of each TKI by HPLC literature,^14^, ^19^, ^20^, ^21^, ^22^, ^23^, ^24^, ^25^ and simultaneous quantification of TKIs with other analytes by LC–MS/MS has also been reported,^14^, ^15^, ^16^, ^17^, ^18^ this method is more efficient. This simultaneous measurement has the advantage that TDM can be performed quickly in the same measurement run, without the need to change the HPLC conditions for each drug when measuring the concentrations of several patients administered with four different TKIs.

The gradient conditions were optimized to separate and detect the peaks of the four TKIs without interference. The organic solvent ratio (B pump) started at 30%, and the binary gradient was adjusted. Previous studies have reported longer retention times (14.5 min for dasatinib,^24^ 34 min for nilotinib,^20^ 15 min for bosutinib,^25^ and 14 min for ponatinib^21^); however, this method can separate the four analytes within 10 min in the following order: dasatinib, bosutinib, ponatinib, and nilotinib. The shorter run time could be more useful for serum concentration analysis of multiple patients administered with different TKIs in clinical practice.

The validation study met FDA guidelines. Good extraction recoveries (80.7%–98.5%) were obtained for the four TKIs. This study demonstrated a recovery rate (Table 3) similar to or higher than those reported in previous studies using similar SPE methods (recovery rates of 74%–76% for dasatinib,^20^ 72%–78% for nilotinib,^20^ 84%–85% for bosutinib,^25^ and 78%–88% for ponatinib^19^). Although the SPE pretreatment method was the same as the one used in previous studies, our use of 2% phosphoric acid as a dilution solvent for the serum samples may have contributed to the higher recovery rate. The accuracy and precision values for dasatinib (2–500 ng/ml) were within ±14.3% and 17.9%, nilotinib values (100–5000 ng/ml) were within ±14.4% and 7.3%, bosutinib values (10–500 ng/ml) were within ±12.8% and 7.3%, and ponatinib values (10–500 ng/ml) were within ±11.9% and 10.1%, respectively. On the other hand, the accuracy and precision values in the previous single analyte assays for dasatinib (10–1000 ng/ml) were within ±4.1% and 15.8%,^20^ the values for nilotinib (10–5000 ng/ml) were within ±10.5% and 10.4%,^20^ the values for bosutinib (25–1500 ng/ml) were within ±10.2% and 8.7%,^25^ and the values for ponatinib (1–250 ng/ml) were within ±9.0% and 10.8%.^19^ The simultaneous measurement of multiple drugs and concentration range of the calibration curves resulted in similar or higher accuracy and precision than previous assays; however, they all met the criteria of FDA's validation guideline. The CV values from the dilution integrity experiments were below 6.4% and accuracy was in the range 85.3%–99.0%, which are consistent with the criteria of the validation guidelines of the FDA. Clinical samples exceeding the calibration range after the first analysis were reanalyzed after diluting them using blank serum up to a five‐fold dilution based on the dilution integrity experiments.

Good calibration curve linearity was obtained for the four analytes (0.987 ≤ *R* ^2^ ≤ 0.998). Measurement of each analyte at a specific wavelength using the PDA detector resulted in a linear calibration curve and high sensitivity; however, the gradient program for simultaneous measurement caused a baseline drift, resulting in poor concentration analysis. Therefore, the LLOQ for dasatinib (2.0 ng/ml) was set to a higher value than that used in previous HPLC assays (0.5 ng/ml).^27^ Although internal standards are generally used in the analysis of clinical samples, no internal standards were used in this assay method. This method met the FDA validation criteria; however, since spiked serum samples tend to have significantly fewer patient‐specific interferences, a larger sampling of true patient specimens is necessary to better interpret the experimental variability of clinical sample analyses.

The results of the stability experiments were consistent with those of previous reports;^21^, ^28^ the stability of the serum samples in an autosampler at −4°C for 24 h enables the quantification of multiple samples.

The calibration range of each analyte was determined based on patients' initial and steady‐state trough and peak concentrations reported in previous studies.^11^, ^14^, ^15^, ^19^ The therapeutic efficacy of the dosage adjustment of each drug based on target blood concentrations was confirmed. The range of the calibration curve covered the specific TDM concentrations for dasatinib (C_0_ < 4.3 ng/ml and C_max_ > 50 ng/ml) and nilotinib (C_0_ = 800 ng/ml).^9^, ^13^ The clinical applicability of the validated HPLC‐PDA method for dasatinib, nilotinib, and bosutinib was confirmed using patient samples. In all the clinical samples, the serum concentrations were within the range of the calibration curve. This HPLC‐PDA method can be applied to the TDM of the four TKIs for CML patients, enabling the construction of an optimal dosing regimen.

## 5 CONCLUSIONS

We established a novel and efficient method for the simultaneous quantification of dasatinib, nilotinib, bosutinib, and ponatinib in serum using HPLC‐PDA. We believe that the method could be used to increase the efficiency of TDM for the four TKIs in patients with CML in clinical practice. The quantification method has been applied to an ongoing clinical trial on the pharmacokinetics of the four TKIs in patients with CML.

## AUTHOR CONTRIBUTION

All authors contributed to the study conception and design. Material preparation and data collection were performed by Yuta Yokoyama, Eiji Nozawa, Miho Morita, Emi Ishikawa, Takehiko Mori, Masatoshi Sakurai, Taku Kikuchi, Eri Matsuki, and Rie Yamazaki. The data were analyzed and interpreted by Yuta Yokoyama, Eiji Nozawa, Miho Morita, Emi Ishikawa, Takehiko Mori, Rie Yamazaki, Sayo Suzuki, and Tomonori Nakamura. Yuta Yokoyama, and Eiji Nozawa wrote the first draft of the manuscript, and previous versions of the manuscript were critically revised by Emi Ishikawa, Takehiko Mori, Eri Matsuki, Keisuke Kataoka, Sayo Suzuki, and Tomonori Nakamura. All authors read and approved the final manuscript.

## FUNDING INFORMATION

This research did not receive any specific grant from funding agencies in the public, commercial, or not‐for‐profit sectors.

## CONFLICT OF INTEREST

The authors report no conflicts of interest.

## Data Availability

The data that support the findings of this study are available from the corresponding author upon reasonable request. Due to the nature of this research, participants of this study did not agree for their clinical data to be shared publicly, so supporting data is not available.
